# Norepinephrine promotes oxidative stress in vascular adventitial fibroblasts via PKC/NFκB-mediated NOX2 upregulation

**DOI:** 10.1080/13510002.2025.2494314

**Published:** 2025-04-23

**Authors:** Yi-Ming Wang, Hong-Ke Dong, Min Dai, Jing-Xiao Wang, Xiao-Yu Xu, Guo-Qing Zhu, Xiu-Zhen Li

**Affiliations:** a Department of Physiology, Nanjing Medical University, Nanjing, People’s Republic of China; b Department of Cardiology, The Second Affiliated Hospital of Nanjing Medical University, Nanjing, People’s Republic of China

**Keywords:** Norepinephrine, vascular adventitial fibroblasts, oxidative stress, NADPH oxidase, hypertension, proliferation, migration, vascular remodeling

## Abstract

**Background:**

Sympathetic overactivity is closely associated with vascular remodeling. Sympathetic fibers dominantly innervate the adventitia of arteries rather than tunica media. Vascular adventitial fibroblasts (VAFs) play crucial roles in vascular remodeling. However, the link between sympathetic overactivity and VAF proliferation and migration is unknown.

**Methods:**

Primary VAFs were isolated from the thoracic aorta of spontaneously hypertensive rats and Wistar–Kyoto rats. Norepinephrine (NE) bitartrate monohydrate was applied to VAFs to simulate the sympathetic overactivity.

**Results:**

NE increased NADPH oxidase (NOX) 2 expression and superoxide level, which were almost abolished by NOX2 inhibitor GSK2795039 or α-adrenoceptor antagonist prazosin, but not significantly affected by NOX1 inhibitor ML171, NOX4 inhibitor GLX351322 or β-adrenoceptor antagonist propranolol. Superoxide scavenger tempol or NOX2 inhibitor GSK2795039 attenuated NE-induced VAF proliferation and migration. NE promoted protein kinase C (PKC) phosphorylation and NFκB-p65 nuclear translocation. Either PKC inhibitor Go6983 or NFκB inhibitor BAY11-7082 attenuated NE-induced NOX activation, NOX2 upregulation, superoxide production, proliferation and migration.

**Conclusion:**

NE promotes oxidative stress by α-receptor/PKC/NFκB-mediated NOX2 upregulation, which contributes to proliferation and migration of VAFs.

## 1. Introduction

Chronic excessive sympathetic activation is an important pathogenic factor for hypertension and related major cardiovascular events [1]. The sympathetic nerve regulates cardiovascular activity, mainly by releasing norepinephrine (NE), and the main source of circulating NE is sympathetic activity [2]. The sympathetic outflow is correlated with circulating NE levels and the severity of hypertension [3,4]. Hypertensive patients with a higher density of sympathetic innervation in the adventitia of the arterioles are more prone to arteriolar damage [5]. The kidney is innervated by sympathetic nerves [6], and renal denervation was used to treat resistant hypertension [7]. Inhibition of excessive sympathetic activation not only attenuates hypertension but also alleviates vascular remodeling [8–10]. Intervention of sympathetic activity is a strategy for the treatment of hypertension [11,12]. Superior cervical ganglionectomy attenuates local vascular remodeling in the ganglion-innervated arteries without significant effects on blood pressure in spontaneously hypertensive rats (SHR), and sympathetic overactivity in hypertension is an independent pathogenic factor for vascular remodeling [13]. The mechanisms of sympathetic overactivity in vascular remodeling are not well known [14].

Phenotype transformation of vascular smooth muscle cells (VSMCs) is associated with vascular remodeling in vascular diseases including hypertension [15]. However, sympathetic fibers mainly innervate the adventitia of arteries [16], suggesting that the adventitia may be the main target of sympathetic activity and NE. Adventitia is a signal analysis and processing center of the artery, and serves as a master regulator of vascular function and structure [17,18]. Vascular adventitial fibroblasts (VAFs) play crucial roles in vascular remodeling [19,20]. Extracellular vesicles (EVs) from VAFs of SHR promote VSMC proliferation, migration and vascular remodeling [21,22]. NE stimulates EV release and the EVs-dependent angiotensin-converting enzyme (ACE) transfer, causing VSMC proliferation in SHR [23]. However, the mechanism of sympathetic activity or NE in regulating VAFs remains unknown.

Reactive oxygen species (ROS) contribute to regulating proliferation and cell cycle [24]. NADPH oxidase (NOX) is a primary source of ROS in vessels, and NOX expression and activity are increased in hypertension [25]. ROS promotes VSMC phenotypic transformation including proliferation and migration [26]. Inhibition of sympathetic overactivity in SHR attenuates oxidative stress and vascular remodeling [9,13]. We speculated that ROS might mediate the effects of NE on VAF proliferation and migration. This study is designed to determine the underlying mechanism of NE in regulating VAF proliferation and migration in normotensive and hypertensive rats.

## 2. Materials and methods

### 2.1. Primary VAFs

SHR is a genetic hypertension model initially bred from Wistar–Kyoto rats (WKY), and the normotensive WKY strain is often used as a control of SHR. Primary VAFs were prepared from the thoracic aorta of male WKY and SHR ages at 8 weeks. The rats were obtained from Vital River Laboratory Animal Technology Co. Ltd (Beijing, China). Systolic blood pressure (SBP) of WKY was higher than 140 mmHg, and that of SHR less than 160 mmHg was excluded from this study [21]. Experiments were approved by the Experimental Animal Care and Use Committee of Nanjing Medical University (No. IACUC-2407020). After an intravenous injection of pentobarbital sodium (150 mg/kg), the thoracic aorta was isolated and perivascular fat tissues were removed quickly. Adventitia was peeled off and then treated with DMEM containing 0.2% collagenase-II for 15–30 min. After centrifugation, the precipitated cells were suspended in the DMEM containing 10% fetal bovine serum (FBS) and then cultured. The VAFs were identified as we previously reported [23], and the cells at 3–5 generations were used in the present study.

### 2.2. Examination of cell proliferation and migration

VAF proliferation was examined with a cell counting kit-8 (CCK8) kit and EdU incorporation assay [27]. For the CCK8 assay, absorbance at 450 nm was measured with a microplate reader. For the EdU incorporation assay, Hoechst33342 was used to label the nuclei of VAFs. Cell nucleus counting was performed in six fluorescence microscope images, with a range of approximately 100–300 nuclei in each image. The percentage of EdU-positive cells was calculated. VAF migration was examined with Boyden chamber assay and wound healing assay [22]. For the Boyden chamber assay, the migrated cells were stained with 1% crystal violet. For the wound healing assay, the images were obtained at 0 and 24 h.

### 2.3. Superoxide and NOX activity measurements

Superoxide production and NOX activity were examined with the Lucigenin-derived chemiluminescence method, as previously reported [28,29]. For the measurement of superoxide production, photon emission was triggered by dark-adapted lucigenin (5 μM). For the measurement of NOX activity, photon emission was initiated by the application of NADPH (100 μM) and dark-adapted lucigenin (5 μM). Background values were obtained by measuring buffer-containing lucigenin (5 µM). Average values were calculated by measuring 10 times within 10 min, and expressed in mean light unit/min/mg protein.

### 2.4. Dihydroethidium fluorescence

Intracellular ROS in VAFs was examined with dihydroethidium (DHE) fluorescence staining [9]. The VAFs at a density of approximately 3 × 10^5^ cells/mL were added into 6-well plates containing PBS with 10 μM of DHE at 37°C for 30 min. The fluorescence was detected using fluorescence microscopy at 518 nm for excitation, and 605 nm for emission. Hoechst33342 was used to show the nuclei of the cells.

### 2.5. Immunofluorescence analysis

Cytoplasmic and nuclei NFκB-p65 in VAFs were detected by immunofluorescence staining. VAFs were incubated with NFκB-p65 antibody (1:100) at 4°C overnight. CY3-conjugated goat anti-rabbit IgG (1:200) was used as the second antibody. The nuclei of VAFs were stained with DAPI (1:1000). Images were collected with fluorescence microscopy.

### 2.6. Western blotting

VAFs were homogenized in lysis buffer. Proteins were separated with 10% SDS-PAGE and transferred to PVDF membrane. The membrane was incubated with the first antibody overnight at 4°C, and with the secondary antibody conjugated with HRP at room temperature for 1 h.

### 2.7. Chemicals and antibodies

Norepinephrine bitartrate monohydrate, prazosin, propranolol, tempol, ML171, GSK2795039, GLX351322, BAY11-7082 and Go 6983 were bought from MCE (Monmouth Junction, NJ, USA). CY3-conjugated goat anti-rabbit IgG was obtained from Servicebio (Wuhan, Hubei, China). NOX1, NOX2, NOX4 and β-actin antibodies were obtained from Proteintech (Wuhan, Hubei, China). NFκB-p65, PKC-pan and phospho-PKC-pan (Thr497) antibodies were purchased from Affinity (Changzhou, Jiangsu, China).

### 2.8. Statistics

Studies were carried out randomly and double-blindly. The number of each group was the independent sample number. IBM SPSS Statistics (Version 24.0) was used for statistical analyses, and SigmaPlot (Version 14.0) was used to generate graphs. Data were expressed as mean ± SD. The distribution of data was checked by the Shapiro–Wilk test. All data showed normal distribution. One-way or two-way ANOVA followed by the Bonferroni test was used for data analysis. *P* < 0.05 was considered to be significantly different.

## 3. Results

### 3.1. Effects of NE on oxidative stress

**Figure 1.**
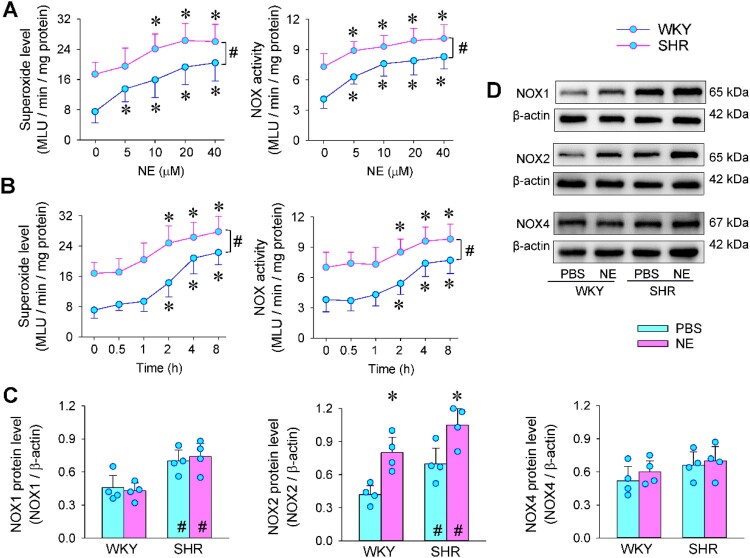
Effects of norepinephrine (NE) on oxidative stress in VAFs of WKY and SHR. (a) dose effects of different concentrations of NE (0, 5, 10, 20 or 40 μM) on superoxide level and NOX activity. The measurements were made 4 h after the application of PBS or NE. (b) time effects of NE (20 μM) on superoxide level and NOX activity. (c) bar graphs showing the NOX1, NOX2 and NOX4 protein expressions. (d) representative images showing the NOX1, NOX2 and NOX4 protein expressions. The measurements were made 4 h after the application of PBS or NE. Values are mean ± SD. Two-way ANOVA followed by Bonferroni post hoc test. *P < 0.05 vs PBS; #*P* < 0.05 vs WKY. *n* = 6 in (a and b). *n* = 4 in (c).

Superoxide and NOX activity in VAFs were upregulated in SHR compared with WKY. NE increased superoxide level and NOX activity in a concentration-dependent manner in VAFs of WKY and SHR. Significant effects were observed at concentrations exceeding 5 μM or 10 μM (Figure 1(a)). The effects started at 2 h after application of 20 μM of NE and lasted at least 8 h (Figure 1(b)). According to the curves of concentration effects and time effects, 20 μM of NE at 4 h was used for the present study. NOX1 and NOX2 protein levels in the VAFs of SHR were higher than those of WKY, but there was no significant difference in NOX4 protein level between WKY and SHR. NE promoted NOX2 expression in both WKY and SHR rather than NOX1 and NOX4 expressions (Figure 1(c and d)).

### 3.2. Roles of NOX2 in NE-induced oxidative stress

**Figure 2.**
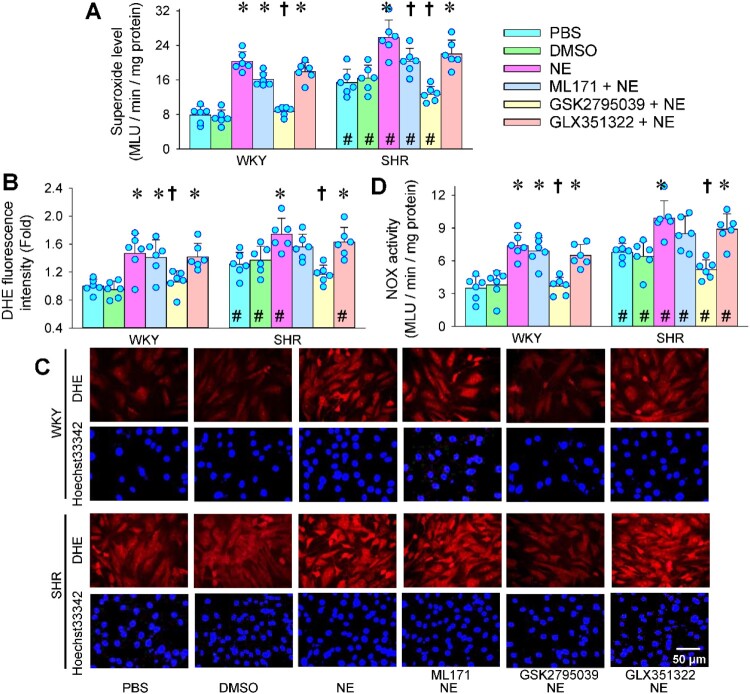
Effects of selective NOX inhibitor on NE-induced oxidative stress in VAFs of WKY and SHR. NE (20 μM) was administered 1 h after the application of NOX1 inhibitor ML171 (10 μM), NOX2 inhibitor GSK2795039 (25 μM), or NOX4 inhibitor GLX351322 (10 μM). Measurements were made 4 h after the application of NE. (a) superoxide level. (b) bar graph showing the relative DHE fluorescence intensity. (c) representative images showing DHE fluorescent staining (red) of VAFs. Nuclei were stained with Hoechst33342 (blue). (d) NOX activity. Two-way ANOVA followed by Bonferroni post hoc test. Values are mean ± SD. *P < 0.05 vs PBS or DMSO; †*P* < 0.05 vs NE alone; #*P* < 0.05 vs WKY. *n* = 6.

Selective NOX2 inhibitor GSK2795039 prevented NE-induced superoxide production in VAFs, but selective NOX1 inhibitor ML171 and NOX4 inhibitor GLX351322 failed to affect the NE-induced superoxide production (Figure 2(a)). The findings were further confirmed by the changes in DHE fluorescence intensity (Figure 2(b and c)). Similarly, the NE-induced increase in NOX activity was prevented by the NOX2 inhibitor rather than the NOX1 inhibitor or NOX4 inhibitor (Figure 2(d)). These results suggest that NOX2 upregulation contributes to NE-induced oxidative stress. However, the superoxide level and NOX activity in the NOX2 inhibitor-treated VAFs of SHR were still higher than those of WKY (Figure 2(a–d)), suggesting that the NOX2 upregulation is not the only mechanism for oxidative stress in the VAFs of SHR. On the other hand, these selective inhibitors of NOX1, NOX2 and NOX4 had no significant effects on NOX2 protein expression, suggesting that NE-induced NOX2 upregulation is not secondary to the activity change of NOXs (Figure S1).

### 3.3. Effects of α- and β-adrenoceptor antagonists on NE-induced oxidative stress

**Figure 3.**
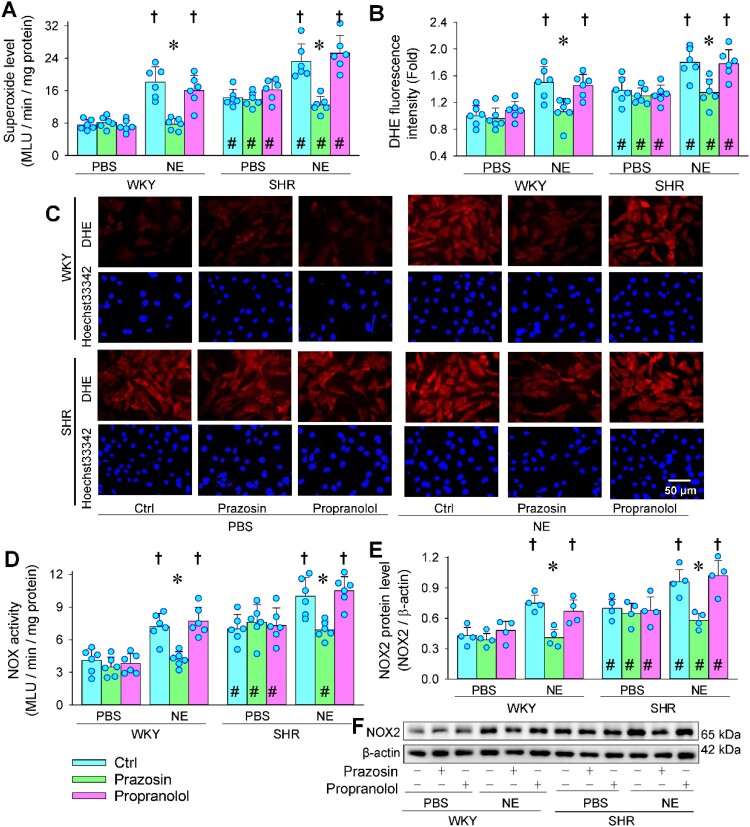
Effects of α- and β-adrenoceptor antagonists on NE-induced oxidative stress in VAFs of WKY and SHR. NE (20 μM) was administered 1 h after the application of α-receptor antagonist prazosin (10 μM) or β-receptor antagonist propranolol (3 μM). Measurements were made 4 h after the application of NE. (a) superoxide level. (b) bar graph showing the relative DHE fluorescence intensity. (c) representative images showing DHE fluorescent staining (red) of VAFs. Nuclei were stained with Hoechst33342 (blue). (d) NOX activity. (e) NOX2 protein expression. (f) representative images showing the NOX2 protein expressions. Values are mean ± SD. Two-way ANOVA followed by Bonferroni post hoc test. *P < 0.05 vs Ctrl; †*P* < 0.05 vs PBS; #*P* < 0.05 vs WKY. *n* = 6 in (a–d); *n* = 4 in e.

Pretreatment with α-adrenoceptor antagonist prazosin prevented the NE-induced increases in the superoxide production and DHE fluorescence intensity in VAFs of WKY and SHR, but β-adrenoceptor antagonist propranolol failed to affect NE-induced oxidative stress (Figure 3(a–c)). Similarly, NE-induced increases in NOX activity and NOX2 expression were abolished by prazosin rather than propranolol (Figure 3(d–f)). The results suggest that α-adrenoceptors in VAFs mediate the NE-induced oxidative stress. Nevertheless, blockage of β-adrenoceptors failed to normalize the NOX2 upregulation and oxidative stress in the VAFs of SHR compared with those of WKY (Figure 3(a–e)).

### 3.4. Roles of oxidative stress in NE-induced VAFs proliferation and migration

**Figure 4.**
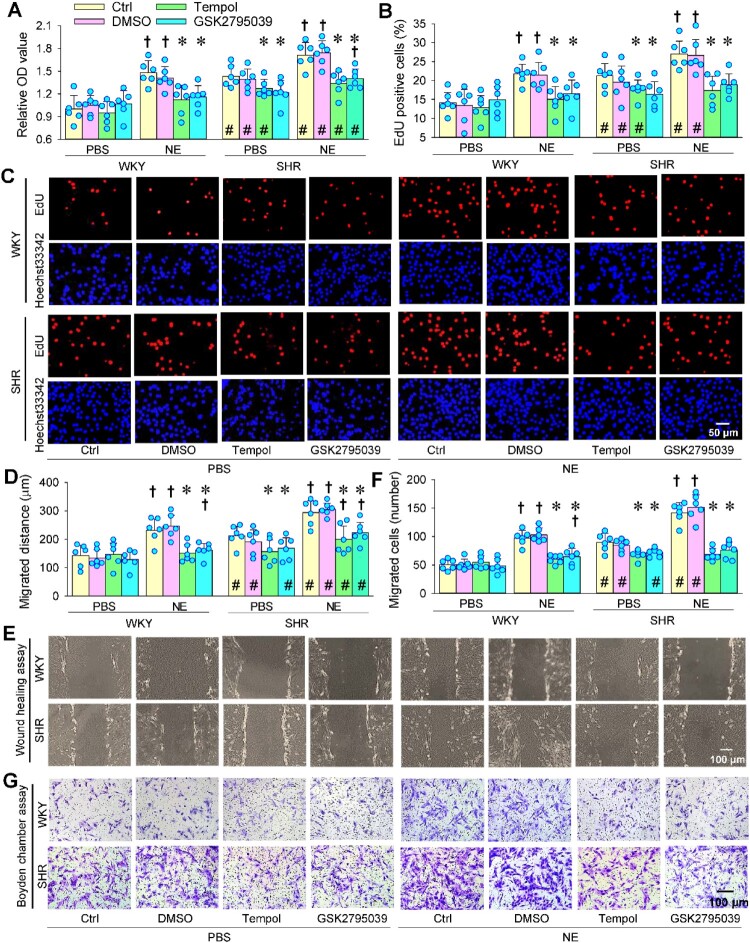
Effects of superoxide scavenger tempol and selective NOX2 inhibitor GSK2795039 on NE-induced VAFs proliferation and migration of WKY and SHR. NE (20 μM) was administered for 1 h after the application of tempol (1 mM) or GSK2795039 (25 μM). Measurements were made 4 h after the application of NE. (a) VAF proliferation was evaluated with CCK-8 kits. (b & c) VAF proliferation was evaluated with the percentage of EdU-positive cells (red). Nuclei were stained with Hoechst33342 (blue). (d & e) VAF migration was evaluated with a wound-healing assay. (f & g) VAFs migration was evaluated with the Boyden chamber assay. Values are mean ± SD. Two-way ANOVA followed by the Bonferroni post hoc test. *P < 0.05 vs Ctrl or 1% DMSO; †*P* < 0.05 vs PBS; #*P* < 0.05 vs WKY. *n* = 6.

NE promoted VAFs’ proliferation of WKY and SHR. Either superoxide scavenger tempol or selective NOX2 inhibitor GSK2795039 abolished the NE-induced VAFs’ proliferation of WKY and SHR (Figure 4(a–c)). NE stimulated VAF migration. Tempol or GSK2795039 prevented the NE-induced VAF migration (Figure 4(d–g)). These results suggest that NOX2-related superoxide production contributes to NE-induced VAF proliferation and migration. Either tempol or GSK2795039 attenuated the VAFs proliferation and migration of SHR, suggesting that oxidative stress partially contributes to the enhanced proliferation and migration in SHR (Figure 4(a–g)).

### 3.5. PKC mediates NE-induced oxidative stress

**Figure 5.**
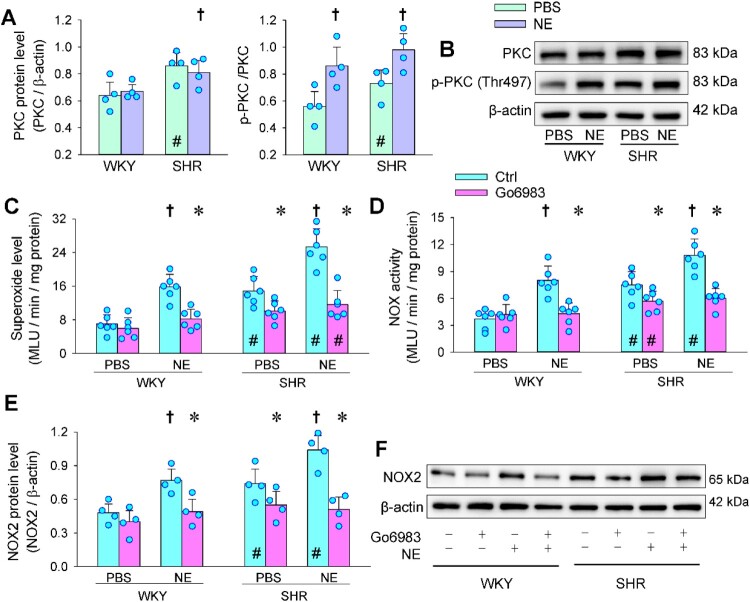
Roles of PKC in mediating the effects of NE on oxidative stress in the VAFs of WKY and SHR. NE (20 μM) was administered for 1 h after the application of PBS or PKC inhibitor Go6983 (3 μM). Measurements were made 4 h after the application of NE. (a) NE-induced PKC phosphorylation. (b) representative images showing the PKC phosphorylation. (c) superoxide level. (d) NOX activity. (e) NOX2 protein expression. (f) representative images showing the NOX2 expression. Values are mean ± SD. *P < 0.05 vs Ctrl; †*P* < 0.05 vs PBS; #*P* < 0.05 vs WKY. *n* = 4 in (a & e); *n* = 6 in (c & d).

It is known that NE activates PKC by acting on α-adrenoceptors in the myocardium and endothelial cells [30,31]. PKC activates NFκB-p65 nuclear translocation [32] and then promotes NOX2 expression [33–36]. Protein kinase C (PKC) was upregulated in the VAFs of SHR, and NE promoted the PKC phosphorylation in WKY and SHR (Figure 5(a and b)). The NE-induced PKC phosphorylation was blocked by α-adrenoceptor antagonist prazosin but was not significantly affected by β-adrenoceptor antagonist propranolol, suggesting that the effects of NE on PKC phosphorylation were mediated by α-adrenoceptors rather than β-adrenoceptors (Figure S2). PKC inhibitor Go6983 only reduced the baseline superoxide level and NOX activity in SHR, but not in WKY. However, Go6983 inhibited the NE-induced increases in the superoxide level and NOX activity in both WKY and SHR (Figure 5(c and d)). Similarly, Go6983 prevented the NE-induced NOX2 upregulation in both WKY and SHR (Figure 5(e and f)). These results suggest that PKC phosphorylation-dependent NOX2 upregulation mediates NE-induced superoxide production.

### 3.6. Transcription factor NFκB is the downstream target of PKC

**Figure 6.**
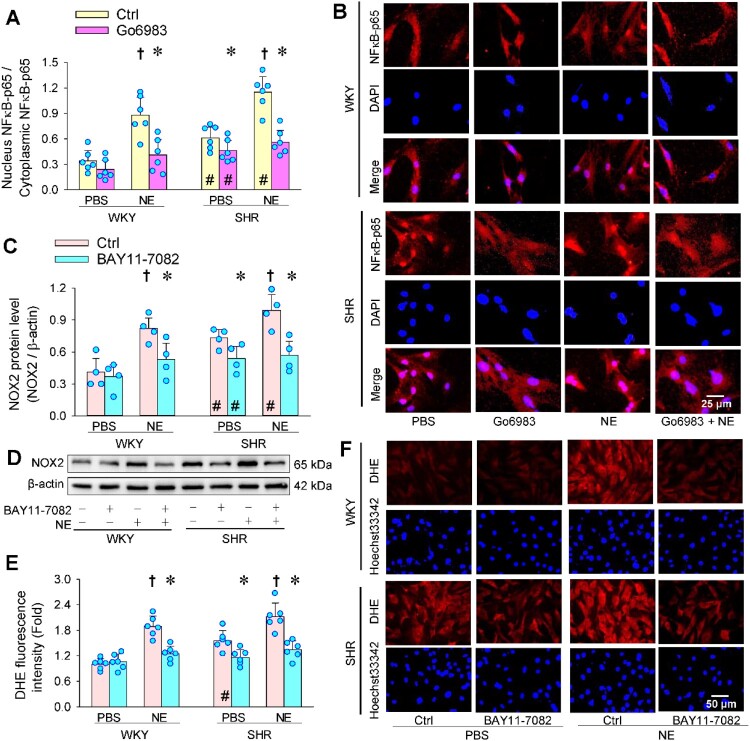
Transcription factor NFκB is the downstream target of PKC in NE-induced oxidative stress in VAFs. (a) effects of PKC inhibitor Go6983 (3 μM) on the ratio of nucleus NFkB-p65 to cytoplasmic NFkB-p65. (b) representative immunofluorescence staining images showing the effects of NE (20 μM) and PKC inhibitor Go6983 (3 μM) on NFκB-p65 levels in cytoplasm and nucleus. Red, NFκB-p65; Blue, DAPI. (c) effects of NFκB inhibitor BAY11-7082 (10 μM) on NE-induced NOX2 protein upregulation. (d) representative images showing the NOX2 expression. (e) DHE staining showing the effects of BAY11-7082 on NE-induced oxidative stress. (f) representative images showing the DHE staining. Values are mean ± SD. Two-way ANOVA followed by Bonferroni post hoc test. *P < 0.05 vs Ctrl; †*P* < 0.05 vs PBS; #*P* < 0.05 vs WKY. *n* = 6 in (a & e); *n* = 4 in c.

Immunofluorescence analysis showed that NE promoted NFκB-p65 nuclear translocation in VAFs of WKY and SHR, which was prevented by PKC inhibitor Go6983. Go6983 only inhibited the enhanced baseline NFκB-p65 nuclear translocation in SHR, but not in WKY (Figure 6(a and b)). These findings were further confirmed by Western blot analysis (Figure S3). BAY11-7082 is a specific NFκB inhibitor, which inhibits the translocation of p65 in the nucleus [37]. Pretreatment with BAY11-7082 abolished the NE-induced NOX2 upregulation and superoxide production of WKY and SHR. However, BAY11-7082 only inhibited the baseline NOX2 expression (Figure 6(c and d)) and superoxide production (Figure 6(e and f)) in SHR, but not in WKY. The results suggest that NE-induced oxidative stress through PKC-dependent NFκB-p65 nuclear translocation.

### 3.7. PKC/NFκB signaling in NE-induced VAF proliferation and migration

**Figure 7.**
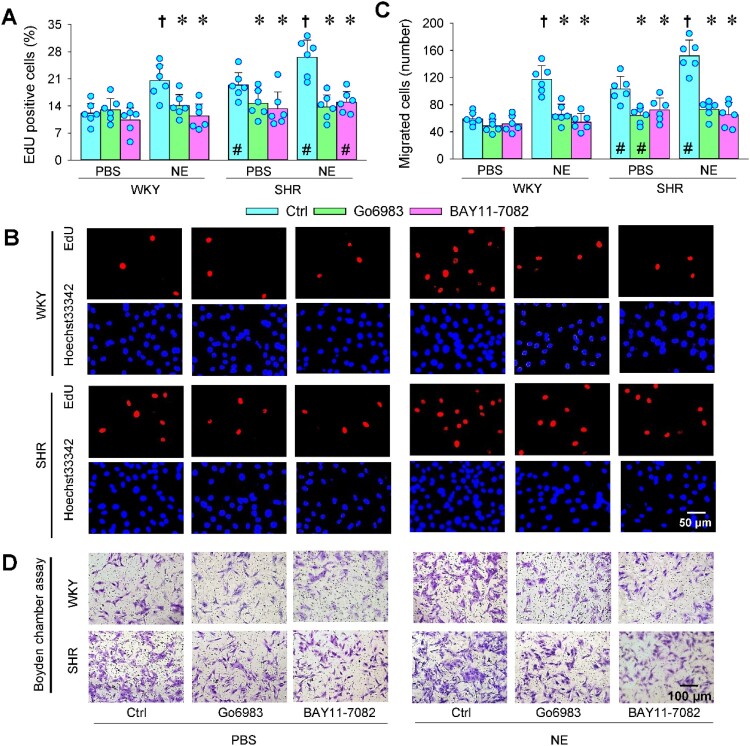
Effects of PKC inhibitor Go6983 and NFκB inhibitor BAY11-7082 on NE-induced VAFs proliferation and migration of WKY and SHR. NE (20 μM) was administered for 1 h after the application of Go6983 (3 μM) or BAY11-7082 (10 μM). Measurements were made 4 h after application of NE. (a & b) VAF proliferation was evaluated with EdU-positive cells (red). Nuclei were stained with Hoechst33342 (blue). (c & d) VAFs migration was evaluated with Boyden chamber assay. Data are mean ± SD. *P < 0.05 vs Ctrl; †*P* < 0.05 vs PBS; #*P* < 0.05 vs WKY. *n* = 6.

Either PKC inhibitor Go6983 or NFκB inhibitor BAY11-7082 inhibited the baseline VAFs proliferation of SHR, but not in WKY. However, the role of NE in stimulating VAF proliferation was prevented by Go6983 or BAY11-7082 in both WKY and SHR (Figure 7(a and b)). Similarly, either Go6983 or BAY11-7082 only inhibited VAFs migration of SHR and abolished NE-induced VAFs migration of WKY and SHR (Figure 7(c and d)). The results suggest that NE promotes VAF proliferation and migration by activating the PKC/NFκB signaling pathway.

## 4. Discussion

Persistent excessive sympathetic activation is an important pathogenic factor for hypertension [1]. Sympathetic fibers dominantly innervate the adventitia of the artery rather than the medium [16]. VAF is the main cellular component of vascular adventitia [17] and increased ROS in VAFs is an initiator and harbinger of vascular remodeling [38]. We have found that increased NE stimulates EV release from VAFs of SHR, causing VSMC proliferation [20]. The main novel findings in this study are that increased NE promotes oxidative stress of VAFs via the α-receptor-mediated PKC/NFκB signaling pathway, which contributes to VAFs proliferation and migration in WKY and SHR.

Accumulated evidence has shown that inhibition of sympathetic overactivity attenuates vascular remodeling and hypertension. It was not clear whether the attenuation of vascular remodeling was directly caused by sympathetic inhibition or secondary to attenuated hypertension in previous studies [8–10]. We recently showed that removing local sympathetic innervation in SHR reduced corresponding vascular remodeling without significant changes in blood pressure, indicating that sympathetic overactivity is an independent etiological factor for vascular remodeling [13]. NE caused oxidative stress via acting on adrenergic α-receptors, and the NE-induced oxidative stress contributed to the VAFs proliferation and migration. The results suggest that excessive NE might be involved in the vascular remodeling of SHR, which was supported by our recent findings that NE promoted EV release from VAFs and then stimulated VSMC proliferation [20]. The in vitro findings further confirmed that sympathetic overactivity directly promoted vascular remodeling in SHR [13].

ROS regulates vascular function and structure as signaling molecules, and excessive ROS production leads to oxidative stress and vascular remodeling in hypertension [39]. NOX1 and NOX2 are the main source of superoxide anions in the arteries [40]. NOX4 is the main source of hydrogen peroxide and is involved in various cellular biological processes [41]. We found that the effects of NE in VAFs appeared after 2 h, rather than immediately, suggesting that NE-induced oxidative stress may be associated with the NOX upregulation, rather than direct NOX activation. Although NOX1, NOX2 and NOX4 expressions in VAFs were upregulated in SHR, NE only promoted NOX2 expression. The NE-induced increases in superoxide level and NOX activity were prevented by selective NOX2 inhibitor GSK2795039 or α-receptor antagonist prazosin, but not affected by NOX1 inhibitor ML171, NOX4 inhibitor GLX351322 or β-adrenoceptor antagonist propranolol. These results indicate that ɑ-receptor-mediated NOX2 upregulation is responsible for the effects of NE on the increases in superoxide level and NOX activity in VAFs.

NOX2 is one of the target proteins of the NFκB signaling pathway [33,34]. NFκB serves as a transcription factor in inducing the expressions of gp91phox (NOX2), p47phox and p67phox (two regulatory subunits required for NOX2 activation) [35,36]. Endogenous NE activates PKC via acting α-adrenoceptors [30], and blockade of α-adrenoceptors prevents PKC activation [31]. PKC activates NFκB by increasing p65 nuclear translocation and initiating related target gene expression [32]. We found that NE activates the α-adrenoceptors and its downstream PKC/NFκB signaling pathway, leading to the upregulation of NOX2 in VAFs of WKY and SHR.

Either superoxide scavenger or selective NOX2 inhibitor inhibited the role of NE in stimulating VAFs proliferation and migration of WKY and SHR. Furthermore, PKC inhibitor Go6983 or NFκB inhibitor BAY11-7082 prevented the NE-induced VAF proliferation and migration. The results indicate that oxidative stress and PKC/NFκB signaling pathway are responsible for NE-induced VAFs proliferation and migration. It is noted that Go6983 or BAY11-7082 attenuated the baseline NOX2 expression, superoxide production, proliferation and migration in SHR, suggesting that the activation of PKC/NFκB signaling pathway contributes to the oxidative stress and enhanced proliferation and migration in hypertensive rats. However, Go6983 or BAY11-7082 had no significant effects on these baseline values in WKY, which does not mean that the PKC/NFκB signaling pathway is not involved in regulating oxidative stress and enhanced proliferation and migration in physiological conditions. The changes in PKC/NFκB signaling may be counteracted by other compensatory mechanisms, as certain levels of superoxide, proliferation and migration are necessary for normal function. The limitation of this study is that the effects of NE was not examined in animals. However, the deficiencies may be partially addressed by our recent studies that inhibition of sympathetic overactivity in SHR attenuated vascular remodeling and oxidative stress [9,13].

It is known that persistent sympathetic overactivity is an important characteristic of hypertension, and is closely associated with plasma NE levels and the severity of hypertension [3,4]. Oxidative stress is closely associated with vascular remodeling and hypertension [39]. This study showed that NE promoted oxidative stress, cell proliferation and migration, suggesting that intervention of NE-induced oxidative stress may play beneficial roles in attenuating vascular remodeling and hypertension. Renal denervation (RDN) is a catheter-based therapy for resistant hypertension, which reduces sympathetic activity [7]. The antihypertensive effect of RDN may partially be attributed to the attenuated vascular remodeling due to reduced sympathetic activity and NE release.

In summary, increased NE promotes VAFs oxidative stress, which contributes to proliferation and migration in VAFs of SHR. The effect of NE on oxidative stress is attributed to the upregulation of NOX2, which is mediated by the α-receptor/PKC/NFκB signaling pathway. Inhibition of PKC or NFκB attenuates NE-induced oxidative stress, which may have a beneficial effect on alleviating vascular remodeling associated with sympathetic overactivity.

## Authors’ contributions

YMW, GQZ and XZL designed the research. YMW, HKD, MD, JXW and XYX performed the experiments. YMW, GQZ and XZL analyzed the data. GQZ supervised the study. YMW, GQZ and XZL wrote and revised the manuscript.

## Supplementary Material

Online supplmentary data.docx

## Data Availability

Data are available from the corresponding author upon reasonable request.

## References

[1] DeLalio LJ, Sved AF, Stocker SD. Sympathetic nervous system contributions to hypertension: updates and therapeutic relevance. Can J Cardiol. 2020;36(5):712–720. doi:10.1016/j.cjca.2020.03.003 32389344 PMC7534536

[2] Grassi G. Assessment of sympathetic cardiovascular drive in human hypertension: achievements and perspectives. Hypertension. 2009;54(4):690–697. doi:10.1161/HYPERTENSIONAHA.108.119883 19720958

[3] Grassi G, Pisano A, Bolignano D, et al. Sympathetic nerve traffic activation in essential hypertension and its correlates. Systematic reviews and meta-analyses. Hypertension. 2018;72(2):483–491. doi:10.1161/HYPERTENSIONAHA.118.11038 29915014

[4] Oparil S, Acelajado MC, Bakris GL, et al. Hypertension. Nat Rev Dis Primers. 2018;4:18014. doi:10.1038/nrdp.2018.14 29565029 PMC6477925

[5] Mauriello A, Rovella V, Anemona L, et al. Increased sympathetic renal innervation in hemodialysis patients is the anatomical substrate of sympathetic hyperactivity in end-stage renal disease. J Am Heart Assoc. 2015;4(12):e002426. doi:10.1161/JAHA.115.002426 26611731 PMC4845297

[6] Dai M, Li CY, Wang JX, et al. Anatomic and functional evidence for renal autonomic innervation in normotensive and hypertensive rats. Am J Physiol Renal Physiol. 2024;327(5):F885–F898. doi:10.1152/ajprenal.00133.2024 39298550

[7] Salehin S, Karnkowska B, Hamza I, et al. Renal denervation in the management of resistant hypertension: a comprehensive review of literature. Curr Probl Cardiol. 2024;49(2):102137. doi:10.1016/j.cpcardiol.2023.102137 37863457

[8] Dorr O, Liebetrau C, Mollmann H, et al. Beneficial effects of renal sympathetic denervation on cardiovascular inflammation and remodeling in essential hypertension. Clin Res Cardiol. 2015;104(2):175–184. doi:10.1007/s00392-014-0773-4 25326158

[9] Wu LL, Zhang Y, Li XZ, et al. Impact of selective renal afferent denervation on oxidative stress and vascular remodeling in spontaneously hypertensive rats. Antioxidants (Basel). 2022;11(5):1003. doi:10.3390/antiox11051003 35624870 PMC9137540

[10] Li P, Huang PP, Yang Y, et al. Renal sympathetic denervation attenuates hypertension and vascular remodeling in renovascular hypertensive rats. J Appl Physiol. 2017;122(1):121–129. doi:10.1152/japplphysiol.01019.2015 27742806

[11] Guber K, Kirtane AJ. Renal sympathetic denervation for hypertension. Kidney Int Rep. 2022;7(10):2129–2140. doi:10.1016/j.ekir.2022.06.019 36217529 PMC9546727

[12] Seravalle G, Grassi G. Sympathetic nervous system and hypertension: new evidences. Auton Neurosci. 2022;238:102954. doi:10.1016/j.autneu.2022.102954 35151003

[13] Wang JX, Xu XY, Wang YM, et al. Superior cervical ganglionectomy attenuates vascular remodeling in spontaneously hypertensive rats. J Hypertens. 2025;43(2):236–245. doi:10.1097/HJH.0000000000003883 39445597

[14] Rizzoni D, gabiti-Rosei C, De CC. State of the art review: vascular remodeling in hypertension. Am J Hypertens. 2023;36(1):1–13. doi:10.1093/ajh/hpac093 35961002

[15] Qi Y, Dai F, Gu J, et al. Biomarkers in VSMC phenotypic modulation and vascular remodeling. Pharmazie. 2019;74(12):711–714. 31907108 10.1691/ph.2019.9743

[16] Hirst GD, Edwards FR. Sympathetic neuroeffector transmission in arteries and arterioles. Physiol Rev. 1989;69(2):546–604. doi:10.1152/physrev.1989.69.2.546 2467318

[17] Stenmark KR, Yeager ME, El Kasmi KC, et al. The adventitia: essential regulator of vascular wall structure and function. Annu Rev Physiol. 2013;75:23–47. doi:10.1146/annurev-physiol-030212-183802 23216413 PMC3762248

[18] Tinajero MG, Gotlieb AI. Recent developments in vascular adventitial pathobiology: the dynamic adventitia as a complex regulator of vascular disease. Am J Pathol. 2020;190(3):520–534. doi:10.1016/j.ajpath.2019.10.021 31866347

[19] Strauss BH, Rabinovitch M. Adventitial fibroblasts: defining a role in vessel wall remodeling. Am J Respir Cell Mol Biol. 2000;22(1):1–3. doi:10.1165/ajrcmb.22.1.f172 10615057

[20] Ye C, Zheng F, Wu N, et al. Extracellular vesicles in vascular remodeling. Acta Pharmacol Sin. 2022;43(9):2191–2201. doi:10.1038/s41401-021-00846-7 35022541 PMC9433397

[21] Ren XS, Tong Y, Qiu Y, et al. MiR155-5p in adventitial fibroblasts-derived extracellular vesicles inhibits vascular smooth muscle cell proliferation via suppressing angiotensin-converting enzyme expression. J Extracell Vesicles. 2020;9(1):1698795. doi:10.1080/20013078.2019.1698795 31839907 PMC6896498

[22] Tong Y, Ye C, Ren XS, et al. Exosome-mediated transfer of ACE (angiotensin-converting enzyme) from adventitial fibroblasts of spontaneously hypertensive rats promotes vascular smooth muscle cell migration. Hypertension. 2018;72(4):881–888. doi:10.1161/HYPERTENSIONAHA.118.11375 30354715

[23] Ye C, Zheng F, Xu T, et al. Norepinephrine acting on adventitial fibroblasts stimulates vascular smooth muscle cell proliferation via promoting small extracellular vesicle release. Theranostics. 2022;12(10):4718–4733. doi:10.7150/thno.70974 35832088 PMC9254254

[24] Mackova V, Raudenska M, Polanska HH, et al. Navigating the redox landscape: reactive oxygen species in regulation of cell cycle. Redox Rep. 2024;29(1):2371173. doi:10.1080/13510002.2024.2371173 38972297 PMC11637001

[25] Touyz RM, Rios FJ, ves-Lopes R, et al. Oxidative stress: a unifying paradigm in hypertension. Can J Cardiol. 2020;36(5):659–670. doi:10.1016/j.cjca.2020.02.081 32389339 PMC7225748

[26] Badran A, Nasser SA, Mesmar J, et al. Reactive oxygen species: modulators of phenotypic switch of vascular smooth muscle cells. Int J Mol Sci. 2020;21(22):8764. doi:10.3390/ijms21228764 33233489 PMC7699590

[27] Sun HJ, Ren XS, Xiong XQ, et al. NLRP3 inflammasome activation contributes to VSMC phenotypic transformation and proliferation in hypertension. Cell Death Dis. 2017;8(10):e3074. doi:10.1038/cddis.2017.470 28981106 PMC5680591

[28] Sheu ML, Shen CC, Chen YS, et al. Ochratoxin A induces ER stress and apoptosis in mesangial cells via a NADPH oxidase-derived reactive oxygen species-mediated calpain activation pathway. Oncotarget. 2017;8(12):19376–19388. doi:10.18632/oncotarget.14270 28038445 PMC5386691

[29] Wu N, Zheng F, Li N, et al. RND3 attenuates oxidative stress and vascular remodeling in spontaneously hypertensive rat via inhibiting ROCK1 signaling. Redox Biol. 2021;48(12):102204. doi:10.1016/j.redox.2021.102204 34883403 PMC8661704

[30] Obata T. Adenosine production and its interaction with protection of ischemic and reperfusion injury of the myocardium. Life Sci. 2002;71(18):2083–2103. doi:10.1016/S0024-3205(02)01993-8 12204768

[31] Tian T, Yu Q, Yang D, et al. Endothelial a1-adrenergic receptor activation improves cardiac function in septic mice via PKC-ERK/p38MAPK signaling pathway. Int Immunopharmacol. 2024;141:112937. doi:10.1016/j.intimp.2024.112937 39182270

[32] Yuan L, Zhang L, Yao N, et al. Upregulation of UGT1A1 expression by ursolic acid and oleanolic acid via the inhibition of the PKC/NFkB signaling pathway. Phytomedicine. 2021;92:153726. doi:10.1016/j.phymed.2021.153726 34536821

[33] Marullo R, Werner E, Zhang H, et al. HPV16 e6 and E7 proteins induce a chronic oxidative stress response via NOX2 that causes genomic instability and increased susceptibility to DNA damage in head and neck cancer cells. Carcinogenesis. 2015;36(11):1397–1406. doi:10.1093/carcin/bgv126 26354779 PMC4751247

[34] Cruz-Gregorio A, randa-Rivera AK. Redox-sensitive signalling pathways regulated by human papillomavirus in HPV-related cancers. Rev Med Virol. 2021;31(6):e2230. doi:10.1002/rmv.2230 33709497

[35] Anrather J, Racchumi G, Iadecola C. NF-kappaB regulates phagocytic NADPH oxidase by inducing the expression of gp91phox. J Biol Chem. 2006;281(9):5657–5667. doi:10.1074/jbc.M506172200 16407283

[36] Gauss KA, Nelson-Overton LK, Siemsen DW, et al. Role of NF-kappaB in transcriptional regulation of the phagocyte NADPH oxidase by tumor necrosis factor-alpha. J Leukoc Biol. 2007;82(3):729–741. doi:10.1189/jlb.1206735 17537988

[37] Hu S, Luo Q, Cun B, et al. The pharmacological NF-kB inhibitor BAY11-7082 induces cell apoptosis and inhibits the migration of human uveal melanoma cells. Int J Mol Sci. 2012;13(12):15653–15667. doi:10.3390/ijms131215653 23443086 PMC3546654

[38] Haurani MJ, Pagano PJ. Adventitial fibroblast reactive oxygen species as autacrine and paracrine mediators of remodeling: bellwether for vascular disease? Cardiovasc Res. 2007;75(4):679–689. doi:10.1016/j.cardiores.2007.06.016 17689510

[39] Garcia-Redondo AB, Aguado A, Briones AM, et al. NADPH oxidases and vascular remodeling in cardiovascular diseases. Pharmacol Res. 2016;114:110–120. doi:10.1016/j.phrs.2016.10.015 27773825

[40] Drummond GR, Selemidis S, Griendling KK, et al. Combating oxidative stress in vascular disease: NADPH oxidases as therapeutic targets. Nat Rev Drug Discov. 2011;10(6):453–471. doi:10.1038/nrd3403 21629295 PMC3361719

[41] Diaba-Nuhoho P, Mittag J, Brunssen C, et al. The vascular function of resistance arteries depends on NADPH oxidase 4 and is exacerbated by perivascular adipose tissue. Antioxidants (Basel). 2024;13(5):503. doi:10.3390/antiox13050503 38790608 PMC11118120

