# Supplementary material for: Norepinephrine promotes oxidative stress in vascular adventitial fibroblasts via PKC/NFκB-mediated NOX2 upregulation
Source: Redox Rep. 2025 Apr 23;30(1):2494314. doi: 10.1080/13510002.2025.2494314 (PMC12024498; doi:10.1080/13510002.2025.2494314)
Supplement: Online supplmentary data.docx [file YRER_A_2494314_SM2543.docx]

**Online supplementary data**

**
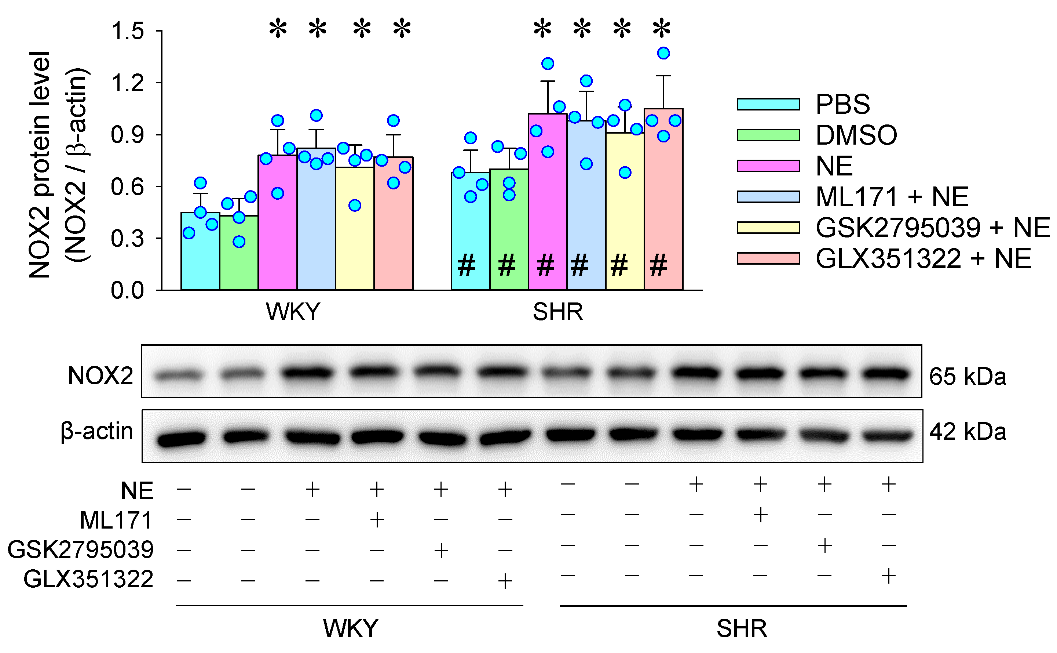
**

Figure S1 Effects of selective NOX inhibitor on NE-induced NOX2 protein expression in VAFs of WKY and SHR. NE (20 μM) was administered 1 h after application of NOX1 inhibitor ML171 (10 μM), NOX2 inhibitor GSK2795039 (25 μM), or NOX4 inhibitor GLX351322 (10 μM). Measurements were made 4 h after application of NE. Two-way ANOVA followed by Bonferroni post hoc test. Values are mean±SD. *P<0.05 vs PBS or DMSO; #P<0.05 vs WKY. n=4.

**
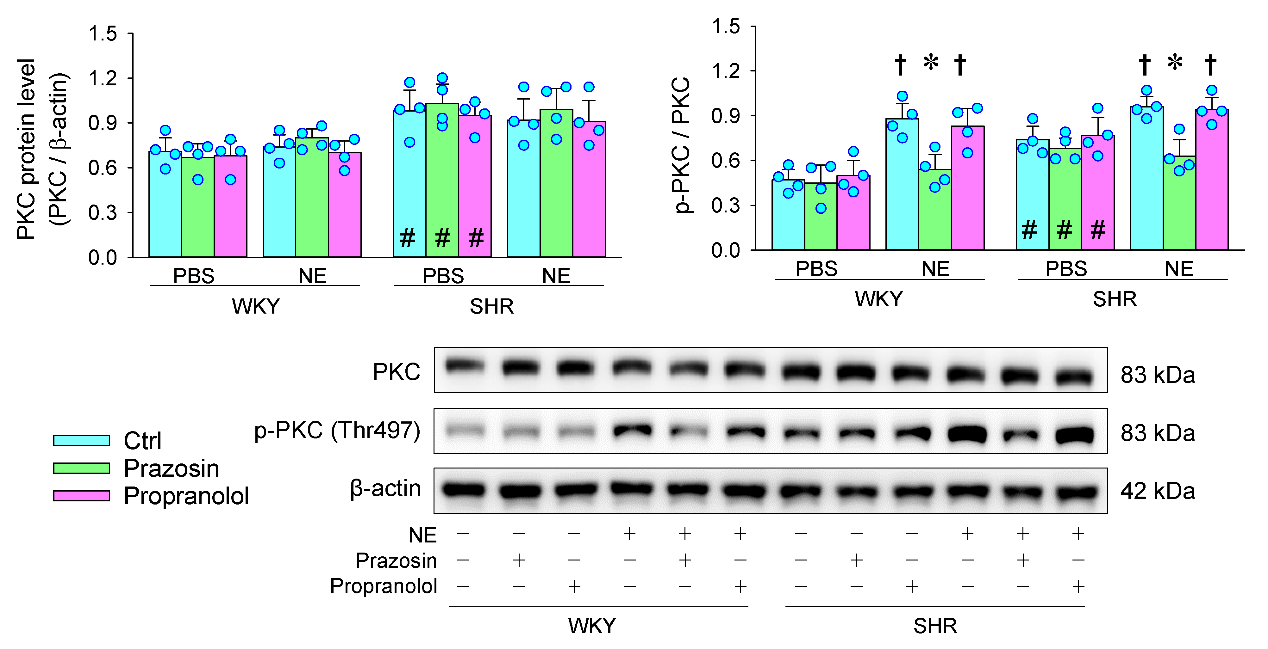
**

Figure S2 Effects of α- and β-receptor antagonists on NE-induced PKC phosphorylation in VAFs of WKY and SHR. NE (20 μM) was administered 1 h after application of α-receptor antagonist prazosin (10 μM) or β-receptor antagonist propranolol (3 μM). Measurements were made 4 h after application of NE. Values are mean±SD. Two-way ANOVA followed by Bonferroni post hoc test. *P<0.05 vs Ctrl; †P<0.05 vs PBS; #P<0.05 vs WKY. n=4.

**
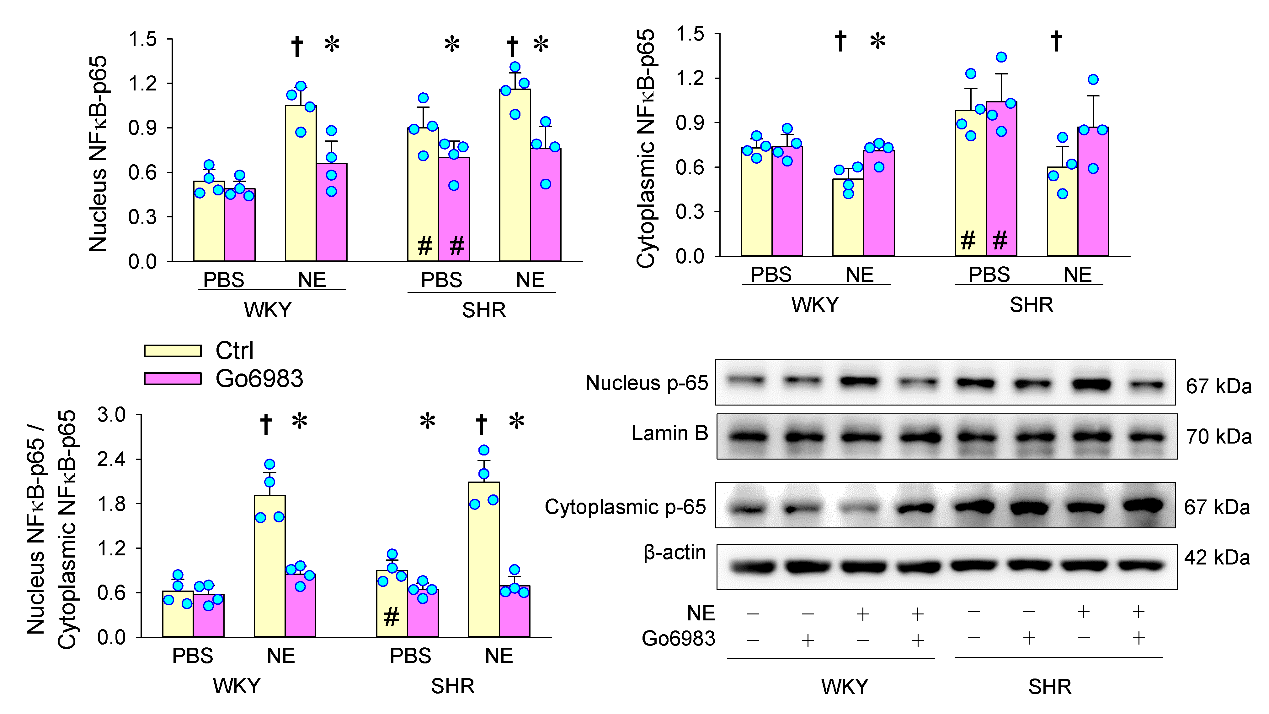
**

Figure S3 Effects of PKC inhibitor Go6983 on NE-induced NFκB-p65 nuclear translocation in VAFs of WKY and SHR. NE (20 μM) was administered 1 h after application of PBS or Go6983 (3 μM). Measurements were made 4 h after application of NE. Values are mean±SD. Two-way ANOVA followed by Bonferroni post hoc test. *P<0.05 vs Ctrl; †P<0.05 vs PBS; #P<0.05 vs WKY. n=4.
